# HELIOS-Stack: A Novel Hybrid Ensemble Learning Approach for Precise Joint Roughness Coefficient Prediction in Rock Discontinuity Analysis

**DOI:** 10.3390/ma18081807

**Published:** 2025-04-15

**Authors:** Ibrahim Haruna Umar, Hang Lin, Hongwei Liu, Rihong Cao

**Affiliations:** 1 School of Resources and Safety Engineering, Central South University, Changsha 410083, China or iharunaumar@kustwudil.edu.ng (I.H.U.); liuhongweicsu@163.com (H.L.); 2 Department of Civil Engineering, Faculty of Engineering, Aliko Dangote University of Science and Technology, Wudil 713101, Kano State, Nigeria

**Keywords:** rock discontinuity, joint roughness coefficient, machine learning, HELIOS-Stack, predictive modeling, Gaussian mixture model (GMM)

## Abstract

Accurate joint roughness coefficient (JRC) estimation is crucial for understanding rock mass mechanical behavior, yet existing predictive models show limitations in capturing complex morphological characteristics of geological surfaces. This study developed an advanced hybrid ensemble learning methodology (HELIOS-Stack) to enhance JRC prediction accuracy by integrating multiple machine learning models and statistical analysis techniques. The research implemented a hybrid ensemble approach combining random forest regression, XGBoost, LightGBM, support vector regression, multilayer perceptron models, and meta-learner using LightGBM as the final estimator. The study analyzed 112 rock samples using eight statistical parameters. Model performance was evaluated against 12 empirical regression models using comprehensive statistical metrics. HELIOS-Stack achieved exceptional accuracy with R^2^ values of 0.9884 (training) and 0.9769 (testing), significantly outperforming traditional empirical models and alternative machine learning models. Also, the HELIOS-Stack statistical evaluation demonstrated superior performance across multiple metrics, including mean absolute error (training: 1.0165, testing: 1.4097) and concordance index (training: 0.99, testing: 0.987). The analysis identified three distinct roughness clusters: high (JRC 16–20), moderate (JRC 8–15), and smooth (JRC 0.4–7). The HELIOS-Stack methodology significantly advances rock discontinuity characterization, establishing a new benchmark for geological surface analysis. This innovative approach offers transformative applications in geotechnical engineering, rock mass stability assessment, and geological modeling through its unprecedented precision in JRC prediction.

## 1. Introduction

Rock discontinuities generally refer to any interruption or break in the continuity of a rock mass, such as faults, joints, bedding planes, or fractures [1]. Rock discontinuities represent critical structural elements in geological formations, profoundly influencing rock masses’ mechanical behavior and stability across various engineering and geological applications [2]. The precise characterization of rock surface roughness has long been challenging in geotechnical engineering, with significant implications for slope stability, underground excavation, dam construction, and geological risk assessment [3]. Traditional methodologies for evaluating rock discontinuity characteristics have been constrained by inherent limitations in capturing complex morphological and mechanical properties of geological surfaces. Joint roughness characteristics play a fundamental role in determining the mechanical behavior and stability of rock masses, with the joint roughness coefficient being a critical parameter in rock engineering applications [4]. Despite its importance, traditional methods of JRC estimation often suffer from subjective assessment and limited accuracy, necessitating more sophisticated approaches to quantification and prediction [5]. The increasing complexity of geotechnical projects demands more precise and reliable methods for characterizing rock joint surfaces, particularly in scenarios where accurate stability assessments are crucial for engineering design and safety considerations [6].

The fundamental limitations of traditional JRC estimation methods manifest in several critical domains. First, conventional approaches rely heavily on subjective visual comparison techniques and simplified empirical models that fail to capture the full complexity of surface morphology [7]. Second, existing methodological frameworks often struggle to accommodate the substantial variability inherent in natural rock formations, resulting in limited predictive reliability [8]. Third, the computational approaches previously employed have been constrained by linear modeling techniques that inadequately represent the nonlinear and multidimensional nature of rock surface characteristics [9].

The integration of artificial intelligence (AI) and machine learning methodologies into geomechanical engineering represents a pivotal paradigm shift in geological and geotechnical research [10]. The evolutionary trajectory of computational approaches in geosciences has progressively demonstrated the transformative potential of advanced algorithmic techniques in addressing complex geological characterization challenges [4]. Deep learning architectures, particularly convolutional neural networks and recurrent neural networks, have emerged as promising computational paradigms in geological surface analysis. Advanced research by Zhao and collaborators illustrated how these sophisticated neural network architectures can effectively interpret three-dimensional geological surface morphologies with unprecedented precision, transcending the limitations of conventional visual and statistical assessment techniques [11]. Ensemble learning methodologies have garnered significant attention in geomechanical engineering research. The integration of multiple machine learning models, such as random forests, gradient boosting machines, and stacking algorithms, has demonstrated superior predictive performance compared to individual algorithmic approaches. Pioneering work by Asteris et al. highlighted the potential of hybrid ensemble techniques in developing more robust and reliable geotechnical predictive models [12]. The application of artificial intelligence in rock discontinuity characterization has witnessed exponential methodological advancements. Researchers like Pradhan and Samui have systematically explored machine learning approaches for rock mass classification, joint roughness coefficient estimation, and geological discontinuity mapping. These investigations have consistently demonstrated the potential of AI methodologies to provide more comprehensive and nuanced geological surface assessments [13].

Emerging research frontiers have increasingly focused on explainable AI techniques, addressing the critical challenge of model interpretability in geomechanical engineering. Local interpretable model-agnostic explanations (LIME) and Shapley additive explanation (SHAP) have emerged as sophisticated methodological approaches to understanding the internal decision-making mechanisms of complex machine learning models, providing unprecedented insights into geological surface characterization processes [14]. Integrating advanced sensing technologies, such as high-resolution 3D scanning, drone-based geological mapping, and advanced sensor networks, has further expanded the computational possibilities in geomechanical engineering. These technological developments have generated increasingly sophisticated datasets that machine learning algorithms can effectively leverage, creating a synergistic relationship between data acquisition and computational analysis. Computational challenges persist in adopting artificial intelligence methodologies in geomechanical engineering. Limited dataset availability, computational complexity, and the inherent variability of geological systems continue to present significant methodological constraints. However, recent advancements in transfer learning, few-shot learning, and generative adversarial networks offer promising approaches to addressing these computational limitations [15].

Recent advances in machine learning and statistical analysis have opened new avenues for improving the accuracy and reliability of JRC predictions. This study introduces HELIOS-Stack (hybrid ensemble learning with integrated optimization and scaling). This novel machine learning approach combines multiple base models in a hierarchical architecture to enhance predictive capability. The methodology integrates five fundamental pillars—random forest regression (RFR), XGBoost (XGB), LightGBM (LGB), support vector regression (SVR), and multilayer perceptron (MLP)—orchestrated through a sophisticated stacking ensemble method, and meta-learner using LightGBM as the final estimator. The research employs a comprehensive analytical framework, incorporating latent class analysis to identify natural groupings in rock discontinuity characteristics, grey correlation analysis to establish parameter hierarchies, and LIME to ensure model interpretability. Through this integrated approach, the study addresses the limitations of conventional empirical methods while providing insights into the relative importance of various roughness parameters in JRC prediction.

**Figure 1:**
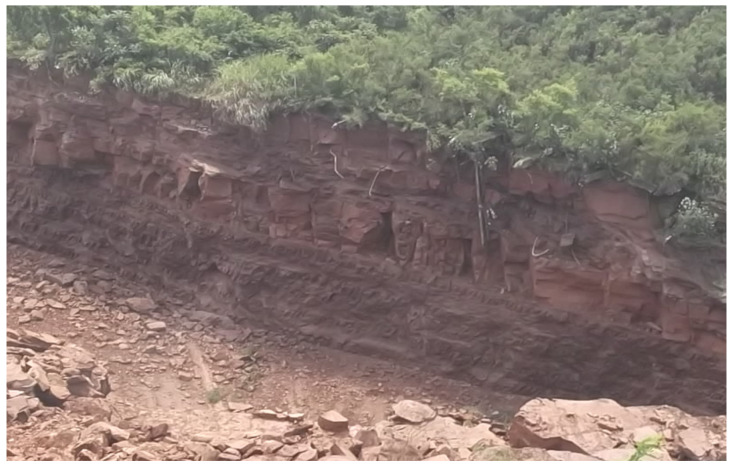
Geological formation with reddish-brown layers of sedimentary rock exposed on a cliff face.

This investigation aims to advance the field of rock mechanics by developing a more robust and accurate method for JRC prediction, comparing its performance against existing empirical models, and establishing a framework for systematically evaluating joint roughness characteristics. The findings have significant implications for geotechnical engineering practice, particularly in applications requiring precise rock mass stability and behavior assessment. Figure 1 shows a rock outcrop with visible discontinuities. These are natural breaks or separations in the rock, such as fractures and joints. These discontinuities are significant in geological studies, as they can influence the stability of the rock mass, affect fluid flow, and provide insights into the area’s geological history and stress conditions. The cliff in Figure 1 has experienced erosion, as evidenced by small caves or indentations in the rock layers. At the top of the cliff, dense vegetation, including bushes and small trees, crowns the formation. Loose rocks and debris are scattered at the base of the cliff. This composition captures a fascinating blend of natural processes like erosion and sedimentation, and it offers an excellent visual for understanding Earth’s geological features.

## 2. Research Methodology

### 2.1. Data Collection

A comprehensive dataset was established by integrating 10 standard profiles supplemented by 102 digitized joint profiles sourced from published literature, particularly referencing the work of Li and Zhang [16]. The compilation encompasses a diverse range of rock discontinuities with projected lengths varying from 72 to 119.6 mm, representing a spectrum from well-interlocked planar configurations to poorly interlocked membrane-covered walls. The geological diversity of the dataset is particularly noteworthy, incorporating specimens from eight distinct rock types: sandstone, limestone, marble, granite, gneiss, slate, dolerite, and siltstone. This selection effectively represents the three primary rock classifications—magmatic, metamorphic, and sedimentary—ensuring broad applicability of the subsequent analyses. The determination of JRC values employed a rigorous back-calculation methodology utilizing direct shear test results in conjunction with the Barton equation. Eight roughness statistical parameters, including structure function of the profile (SF), maximum relative height (R_max_), average relative height (R_ave_), standard deviation of height (SD_h_), root mean square of the first deviation of the profile (Z_2_), average inclination angle (i_ave_), and roughness profile index (R_p_), were systematically calculated for each digitized profile, with comprehensive coordinate information. The dataset underwent strategic partitioning to enhance the predictive model’s robustness and mitigate potential overfitting issues, with 80% (89 samples) allocated to the training set and 20% (23 samples) reserved for model validation. This systematic approach to data organization and model development ensures both comprehensive training and meaningful validation of the proposed predictive framework.

The study utilized a total of 112 rock joint profiles for model development, strategically partitioned into 89 samples (80%) for training and 23 samples (20%) for validation to ensure robustness while mitigating overfitting risks. This split aligns with standard machine learning practices for datasets of moderate size, particularly in geomechanical studies where sample acquisition is often constrained by logistical and experimental complexities. To further validate data consistency, 5-fold cross-validation was integrated into the hyperparameter tuning process for all base models (XGBoost, etc.), ensuring stable performance across different data partitions. The resulting model metrics—such as HELIOS-Stack’s R^2^ on the validation set and minimal systematic bias—demonstrate strong consistency between training and validation phases, reinforcing the dataset’s internal coherence. Additionally, the GCA confirmed high relational consistency between input parameters and JRC targets, underscoring the dataset’s structural reliability.

The pertinent observation regarding the dataset size of 112 rock joint profiles, which indeed represents a moderate sample in the geomechanical studies, and larger datasets could further enhance the generalizability of machine learning models like HELIOS-Stack. However, several factors underscore the robustness and adequacy of the current dataset for the study’s objectives. First, the dataset was meticulously curated to ensure geological diversity, encompassing eight rock types (sandstone, limestone, marble, granite, gneiss, slate, dolerite, and siltstone) across magmatic, metamorphic, and sedimentary classifications. This deliberate inclusion of heterogeneous samples ensures that the model captures a broad spectrum of joint roughness characteristics, mitigating potential biases toward specific lithologies. Second, the profiles were sourced from both laboratory measurements and digitized literature data, ensuring a balanced representation of controlled experimental conditions and real-world variability. While the sample size may appear limited, it aligns with precedent studies in rock mechanics, where empirical JRC estimation often relies on similarly sized datasets due to the labor-intensive nature of joint profiling and shear testing.

### 2.2. Methods Used to Evaluate JRC

Four distinct approaches have played crucial roles in evaluating joint roughness coefficients: visual assessment techniques, mechanical evaluation procedures, statistical analysis, and fractal-based methods. These methodological strategies have significantly advanced our understanding and quantification of JRC.

#### 2.2.1. Visual Comparison Method

The visual comparison method for determining JRC in rock mechanics represents a foundational approach introduced by Barton and Choubey in 1977 [17]. This method fundamentally relies on comparing observed roughness profiles of discontinuity surfaces with standard reference profiles corresponding to established JRC values ranging from 0 to 20 [18]. The technique employs ten standard roughness profiles developed by Barton, with JRC values categorized across distinct intervals, as in Table 1. Implementation of the visual comparison method involves direct visual assessment of the joint surface against these standard profiles [19]. Practitioners evaluate amplitude and wavelength characteristics to select the most similar reference profile, assigning the corresponding JRC value or range. This approach offers several advantages, including rapid field assessment capability, non-destructive evaluation, minimal equipment requirements, and suitability for preliminary investigations [20]. However, the method has inherent limitations worth considering in academic discourse. The subjective nature of the assessment introduces operator dependency, while scale effects are not directly addressed. Furthermore, the method relies on a two-dimensional representation of inherently three-dimensional surfaces, potentially oversimplifying complex geometries. The reliability of assessments can vary significantly between operators, influenced by factors such as experience, lighting conditions, surface weathering effects, and profile length considerations.

Modern academic research has proposed various enhancements to the original visual comparison method. These improvements include developing enhanced reference systems with additional intermediate profiles, region-specific calibration, and digital reference databases. Integrating quantitative support through roughness parameters, statistical analysis frameworks, and digital image processing techniques has refined the methodology. Contemporary adaptations have embraced technological advancement, incorporating three-dimensional scanning, machine learning applications, and automated profile-matching systems. These innovations address traditional limitations while maintaining the method’s simplicity and practicality. For research applications, thorough methodology documentation proves crucial, encompassing profile length, sampling direction, environmental conditions, and assessment protocols. Uncertainty analysis should address confidence intervals, inter-operator variability, scale effects, and spatial variability. Result validation through cross-validation with alternative methods, laboratory testing correlation, and field performance verification ensures robust scientific outcomes.

#### 2.2.2. Mechanical Test Method

The mechanical test method for determining the JRC represents a quantitative approach that directly relates to the fundamental shear behavior of rock discontinuities [21]. This methodology, developed through extensive research and practical applications, provides empirical correlations between measured shear strength parameters and joint roughness characteristics [17]. The underlying principle of the mechanical test method stems from Barton’s empirical criterion, which establishes a relationship between normal stress, joint wall compressive strength, residual friction angle, and the joint roughness coefficient. Through back-calculation using measured peak shear strength values, the JRC can be determined using the well-established Barton equation, where peak shear strength is expressed as a function of these parameters. The Barton equation for peak shear strength and JRC back-calculation can be expressed as in Equation (1), and the solution for JRC is given by Equation (2) [22].

(1) $$\tau_{p}=\sigma_{n}\tan\left[JRC\log_{10}\left(\frac{JCS}{\sigma_{n}}\right)+\phi_{r}\right]$$

(2) $$JRC=\frac{{tan}^{-1}\left(\frac{\tau_{p}}{\sigma_{n}}\right)-\varphi_{r}}{{log}_{10}\left(\frac{JCS}{\sigma_{n}}\right)}$$

where $\tau_{p}\;$ is the peak shear strength, $\sigma_{n}\;$ is the normal stress, JCS is the joint wall compressive strength, and $\varphi_{r}\;$ is the residual friction angle.

This empirical relationship forms the foundation for back-calculating JRC from direct shear test results. The equation demonstrates that joint roughness plays a crucial role in the overall shear strength by contributing to the effective friction angle through a logarithmic function of the stress ratio between joint wall strength and normal stress. The logarithmic term log_10_(JCS/$\sigma_{n}$) represents the scale effect of normal stress on the contribution of surface roughness to shear strength, indicating that roughness has a more significant influence at lower normal stresses.

The method typically involves direct shear testing of rock joint specimens under controlled normal stress conditions in laboratory implementation. The testing procedure requires careful specimen preparation to preserve natural joint surface conditions and precise control of testing parameters, including normal load application, shear displacement rate, and environmental conditions [19]. The specimens must represent field conditions and be sufficiently large to capture meaningful roughness characteristics. The experimental process involves multiple stages of shear testing at different normal stress levels to comprehensively understand the joint’s mechanical behavior. Peak shear strength values obtained from these tests serve as primary data points for JRC back-calculation. The process requires accurate joint wall compressive strength measurement, typically obtained through Schmidt hammer tests or other appropriate strength testing methods [18].

Critical considerations in the mechanical approach include the scale effect, which significantly influences measured shear strength values and consequently affects back-calculated JRC values. The size of test specimens must be carefully selected to represent field-scale joint behavior adequately [23]. Additionally, the method requires attention to specimen damage during testing, as successive shear displacements can alter surface roughness characteristics. The interpretation of mechanical test results demands careful consideration of several factors affecting shear behavior. These include the influence of water content, loading rate effects, and the potential for progressive failure mechanisms [19]. The analysis must account for these variables to obtain reliable JRC values that reflect joint surface characteristics. Advanced implementations of the mechanical method often incorporate sophisticated monitoring systems to track shear displacement, normal displacement and applied loads with high precision. Digital data acquisition systems enable detailed analysis of stress–displacement relationships and provide insights into the progressive development of shear resistance during testing. Statistical treatment of test results is crucial in establishing reliable JRC values. Multiple tests under identical conditions help quantify variability and establish confidence intervals for derived parameters. The methodology must account for inherent variability in natural joint properties and testing conditions.

A significant advantage of the mechanical test method lies in its direct relationship to fundamental joint behavior. Unlike visual or profile measurement techniques, this approach directly incorporates the mechanical response of the joint surface under realistic loading conditions. This provides precious information for engineering applications, where understanding shear strength characteristics is paramount. However, the method presents certain limitations and practical challenges. The requirement for specialized testing equipment and careful specimen preparation makes it more resource-intensive than simpler evaluation methods. Additionally, the destructive nature of testing prevents repeated measurements on the same specimen, necessitating multiple samples for comprehensive characterization. Contemporary research continues to refine the mechanical test method by integrating advanced monitoring techniques and improved data analysis methodologies. Digital image correlation techniques, for instance, enable detailed tracking of surface deformation during shear testing, providing additional insights into the relationship between surface roughness and mechanical behavior. The academic significance of the mechanical test method extends beyond simple JRC determination. The approach provides valuable data for validating theoretical models of joint behavior and developing an improved understanding of scale effects in rock mass behavior. This contributes to the broader field of rock mechanics by establishing quantitative relationships between surface characteristics and mechanical response.

#### 2.2.3. Statistical Parameter Method

**Figure 2:**
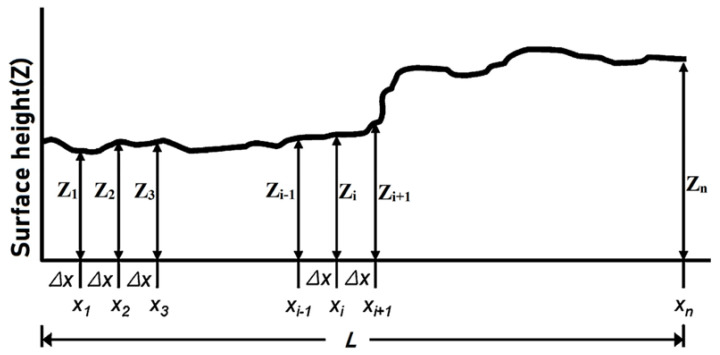
Illustrative diagram depicting the core statistical parameters of a roughness profile adapted from existing research on joint characterization [25].

The statistical parameter method for evaluating joint roughness coefficient is a quantitative approach employing mathematical relationships between measurable surface parameters and joint roughness [22]. This methodology emerged as researchers sought more objective means to characterize joint surface roughness, moving beyond the limitations of visual comparison methods. The fundamental concept relies on establishing correlations between various statistical parameters of measured surface profiles and corresponding JRC values [24]. Figure 2 shows a schematic representation of the roughness profile’s fundamental components, illustrating key statistical parameters as conceptualized in previous research by Kulatilake et al. [25].

Contemporary applications often integrate multiple statistical parameters through multivariate analysis or machine learning techniques, improving the robustness of JRC estimation. These advanced approaches help account for the complex nature of surface roughness and its various manifestations in different rock types. The statistical parameter method continues to evolve with technological advancement and improved understanding of rock joint behavior. While it offers greater objectivity than visual comparison methods, its effective application requires careful consideration of measurement protocols, scale effects, and uncertainty analysis. The method’s mathematical foundation provides a robust basis for roughness characterization while facilitating integration with modern digital measurement and analysis techniques. This quantitative approach to JRC evaluation represents a significant advancement in rock mechanics, providing reproducible results and enabling systematic analysis of joint roughness characteristics. Its continued development through integrating new measurement technologies and analytical methods ensures its relevance in research and practical applications in rock engineering. Table 2 presents a comprehensive compilation of JRC prediction equations developed by various researchers over several decades. These equations utilize different statistical parameters to estimate JRC, demonstrating joint surface characterization’s complexity and multifaceted nature.

The equations above predominantly employ variables such as Z_2_ (root mean square of the first profile deviation), R_p_ (roughness profile index), SF (structure function), and SD (standard deviation), each capturing unique aspects of joint surface morphology. Researchers like Tse and Cruden, Barton and Choubey, and Luo et al. have proposed equations primarily using Z_2_, showing a consistent trend of relating JRC to the statistical variation of surface profile heights [22]. The mathematical formulations reveal intricate relationships between surface characteristics and roughness coefficient. Some equations follow logarithmic transformations, such as Harrison and Rasouli’s approach using the natural logarithm of the structure function, while others employ power law relationships or linear transformations. The coefficients and exponents vary significantly across different studies, reflecting the nuanced interpretations of joint surface roughness. Notably, the equations demonstrate remarkable diversity in their approach to quantifying joint roughness. Some utilize simple linear relationships, while others incorporate more complex mathematical transformations to capture the intricacies of surface topography. This variation underscores the challenges in developing a universally applicable method for JRC estimation. The progression of these equations from 1976 to 2022 illustrates the ongoing scientific refinement of joint roughness characterization. Each iteration represents an attempt to quantify the complex geometric properties of rock joint surfaces more precisely, highlighting the continuous evolution of geomechanical understanding.

#### 2.2.4. Fractal-Based Methods

The application of fractal geometry in characterizing rock joint roughness has emerged as a sophisticated approach that addresses the inherent limitations of traditional JRC estimation methods [34]. The fundamental premise underlying fractal-based analysis stems from the self-affine nature of rock joint surfaces across multiple scales. As a quantitative measure of surface roughness, the fractal dimension provides a mathematical framework for describing the complexity and irregularity of joint profiles [35]. The most commonly employed fractal parameters include the fractal dimension (D) and the roughness amplitude parameter. These parameters can be determined through various mathematical approaches, the most prominent being compass-walking, box-counting, and variogram techniques. The relationship between fractal dimension and JRC has been established through empirical correlations, typically expressed as JRC = a(D − 1) + b, where a and b are empirical constants determined through regression analysis of experimental data. This correlation has demonstrated remarkable consistency across rock types and scaling conditions [36].

One significant advantage of fractal-based methods is their ability to capture the scale-dependent nature of joint roughness. Traditional JRC measurements often suffer from scale-dependent limitations, whereas fractal analysis inherently accounts for the multiscale characteristics of surface roughness. This property makes fractal-based approaches particularly valuable for engineering applications involving varying scales of investigation. Recent advancements in digital photogrammetry and laser scanning technologies have further enhanced the practical implementation of fractal-based methods. These technologies enable high-resolution surface mapping and subsequent fractal analysis, providing more accurate and objective JRC estimations than conventional visual comparison methods. However, certain limitations warrant consideration. The selection of appropriate sampling intervals and profile lengths can significantly influence the calculated fractal parameters. Additionally, the assumption of perfect self-affinity may not always hold true for natural joint surfaces, necessitating careful validation of results.

### 2.3. Analytical Methods

#### 2.3.1. Latent Class Analysis Using GMMs (Using Python Software Foundation, Wilmington, DE, USA)

Gaussian mixture models (GMMs) provide a sophisticated probabilistic approach to latent class analysis, enabling the identification of underlying subpopulations within complex datasets [37,38]. The method employs a mixture of multivariate normal distributions, where the probability density function represents the weighted sum of K component densities, characterized by means μk, covariance matrices Σk, and mixture weights πk. Using expectation-maximization (EM) algorithms, GMMs iteratively estimate these parameters, identifying latent classes through posterior probability assignments [39]. The model’s flexibility allows for capturing heterogeneous data structures, accommodating varying cluster shapes and sizes through different covariance specifications such as spherical, diagonal, or full matrix representations. Model selection criteria like Bayesian information criterion (BIC) or Akaike information criterion (AIC) facilitate optimal class enumeration, enabling researchers to systematically determine the most appropriate number of latent classes that best represent the underlying data structure [40]. For rock discontinuity profile analysis, standardization transforms the disparate measurements (R_ave_, SD_h_, i_ave_, etc.) to comparable scales. The standardization equation is given in Equation (3).

(3) $$z_{ij}=\frac{x_{ij}-\mu_{j}}{\sigma_{j}}$$

where x_ij_ represents the original measurement (e.g., R_ave_ or S_Dh_), μ_j_ is the mean of the parameter, and σ_j_ is its standard deviation. This transformation ensures all parameters contribute equally to subsequent analyses, preventing the dominance of parameters with larger magnitudes.

Rock discontinuity surfaces exhibit complex patterns that often cannot be adequately described by a single statistical distribution [41]. The Gaussian mixture model provides a sophisticated framework for modeling these complex patterns by combining multiple Gaussian distributions [42]. This methodology enables the normalization of the eight roughness parameters to comparable scales, identification of natural groupings in the roughness characteristics, assignment of profiles to distinct roughness classes, and understanding the underlying distribution of roughness parameters. The analysis of the 112 profiles reveals patterns in the roughness parameters that correspond to different JRC values, facilitating automated classification of rock discontinuity surfaces based on their measured characteristics. The GMM approach suits this application because it captures the complex, multimodal nature of roughness parameter distributions in natural rock surfaces. For rock discontinuity classification, the GMM models the probability density of the roughness parameters as a weighted sum of K Gaussian components, which is given in Equation (4).

(4) $$p(x)=\sum_{k=1}^{K}\pi_{k}N(x|\mu_{k},\Sigma_{k})$$

Each component in this mixture is characterized by a multivariate Gaussian distribution that accounts for the correlations between different roughness parameters, as in Equation (5).

(5) $$N(x|\mu_{k},\Sigma_{k})=\frac{1}{{\left(2\pi \right)}^{d/2}|\Sigma_{k}{|}^{1/2}}\exp\left(-\frac{1}{2}{\left(x-\mu_{k}\right)}^{T}\Sigma_{k}^{-1}(x-\mu_{k})\right)$$

where d is the dimensionality of the roughness parameters (8 in this case), μ_k_ is the mean vector, and Σ_k_ is the covariance matrix for component k.

The expectation-maximization algorithm provides an iterative approach to finding the optimal parameters of the Gaussian mixture model. This process is crucial for accurately characterizing the different classes of rock discontinuity profiles. The algorithm alternates between two steps: (a) in the expectation step, it calculates the probability that each profile (i) belongs to each component, as in Equation (6).

(6) $$\gamma_{ik}=\frac{\pi_{k}N(x_{i}|\mu_{k},\Sigma_{k})}{\sum_{j=1}^{K}\pi_{j}N(x_{i}|\mu_{j},\Sigma_{j})}$$

The maximization step then updates the model parameters based on these probabilities and mean vectors update, as in Equation (7), covariance update matrices in Equation (8), and mixing coefficients in Equation (9).

(7) $$\mu_{k}^{new}=\frac{\sum_{i=1}^{N}\gamma_{ik}x_{i}}{\sum_{i=1}^{N}\gamma_{ik}}$$

(8) $$\Sigma_{k}^{new}=\frac{\sum_{i=1}^{N}\gamma_{ik}(x_{i}-\mu_{k}^{new}){\left(x_{i}-\mu_{k}^{new}\right)}^{T}}{\sum_{i=1}^{N}\gamma_{ik}}$$

(9) $$\pi_{k}^{new}=\frac{\sum_{i=1}^{N}\gamma_{ik}}{N}$$

#### 2.3.2. Grey Correlation Analysis Framework

Grey correlation analysis (GCA) is a powerful methodological approach for evaluating complex relationships between multiple variables, particularly in uncertain scenarios and limited information [43]. The technique measures the correlation degree between reference and comparative sequences, utilizing a sophisticated computational framework that quantifies similarity through a grey correlation coefficient γ(k). By calculating the absolute difference between standardized reference and comparative sequences, GCA generates a correlation grade between zero and one, where values closer to one indicate stronger correlational proximity [44]. GCA was employed to investigate the correlative relationships between JRC and various roughness parameters. GCA is particularly suitable for this analysis due to its capacity to handle multiple variables with different units and scales while requiring minimal assumptions about data distribution [45]. The method’s robustness in analyzing small samples and incomplete information systems makes it especially valuable for geotechnical applications. The GCA procedure implemented in this study consists of four primary steps: data preprocessing, deviation sequence calculation, Grey correlation coefficient calculation, and Grey correlation grade calculation. Table 3 shows the correlation criteria for classifying the relationship between input and output parameters.

The initial data series comprises a reference sequence (JRC values) and comparison sequences (roughness parameters), as in Equation (10):

(10) $$X_{0}=\{x_{0}(1),x_{0}(2),\ldots ,x_{0}(n)\},\;X_{i}=\{x_{i}(1),x_{i}(2),\ldots ,x_{i}(n)\}$$

where X_0_ represents the reference sequence, X_i_ represents the comparison sequences (i = 1, 2, …, 8), and n = 112 samples.

Data normalization was performed using min–max scaling to ensure comparability, as in Equation (11):

(11) $$x_{i}^{\prime }(k)=\frac{x_{i}(k)-\min(X_{i})}{\max(X_{i})-\min(X_{i})}$$

where $\;x_{i}^{\prime }$(k) represents the normalized value.

Deviation sequence calculation is the absolute difference between normalized sequences, calculated in Equation (12):

(12) $$\Delta_{i}(k)=\left|x_{0^{\prime }}(k)-x_{i^{\prime }}(k)\right|$$

where Δ_i_(k) is the absolute difference between the normalized reference and comparison sequences at point k, x_0_′(k) is the normalized reference sequence value at point k, and x_i_′(k) is the normalized comparison sequence value at point k.

The grey correlation coefficient is determined using Equation (13):

(13) $$\xi_{i}(k)=\frac{\min_{i}\min_{k}\Delta_{i}(k)+\rho \max_{i}\max_{k}\Delta_{i}(k)}{\Delta_{i}(k)+\rho \max_{i}\max_{k}\Delta_{i}(k)}$$

where ξ_i_(k) is the grey correlation coefficient at point k, ρ is the distinguishing coefficient (typically ρ = 0.5), and max_i_ max_k_ Δ_i_(k) and Δmin_i_ min_k_ Δ_i_(k) are the global maximum and minimum differences, respectively. The distinguishing coefficient ρ = 0.5 was selected based on its optimal balance between differentiation ability and stability, following established practice in grey system theory.

The grey correlation grade γ_i_ for each parameter i is calculated as the average of the grey correlation coefficients in Equation (14):

(14) $$\gamma_{i}=\frac{1}{n}\sum_{k=1}^{n}\xi_{i}(k)$$

where γ_i_ is the grey correlation grade for parameter i, n is the number of samples, and ξ_i_(k) is the grey correlation coefficient at point k.

#### 2.3.3. HELIOS-Stack (Hybrid Ensemble Learning with Integrated Optimization and Scaling) Using Python Software Foundation, Wilmington, DE, USA

The proposed new method, HELIOS-Stack, reflects both the technical sophistication and the hierarchical nature of the model combination while being memorable and professional for academic or industrial applications. The “Stack” suffix emphasizes the stacking ensemble nature of the architecture, differentiating it from other potential HELIOS variants that might be developed for different purposes. Components integrated include base models known as the five pillars (random forest regression (RFR), XGBoost (XGB), LightGBM (LGB), support vector regression (SVR), and multilayer perceptron (MLP)) and meta-learner using LightGBM as the final estimator. Key features include hierarchical learning for multilevel prediction architecture, ensemble optimization using GridSearchCV for each component, integrated scaling for standard scaler preprocessing, orchestrated stack for the coordinated model pipeline, and systematic analysis for the LIME interpretability framework. 

**Figure 3:**
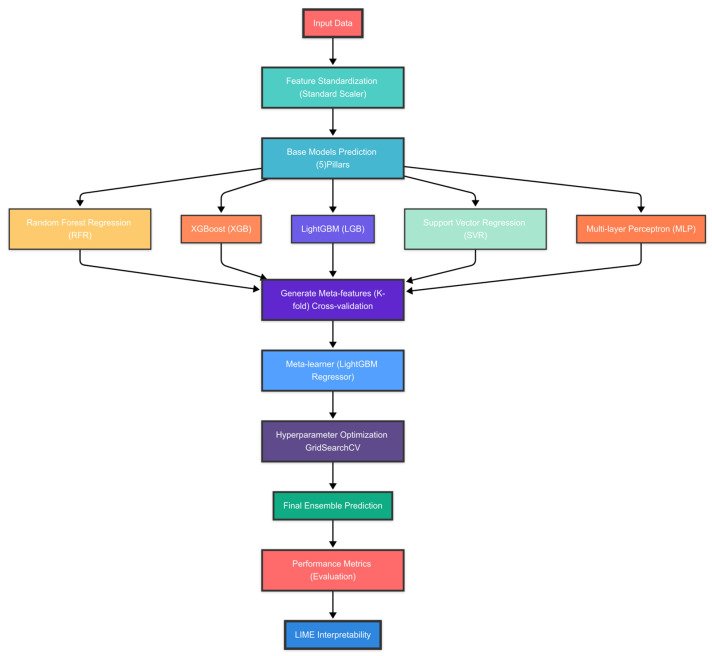
Flowchart of HELIOS-Stack (hybrid ensemble learning with integrated optimization and scaling).

Features are standardized using a standard scaler to ensure all variables are on the same scale as in Equation (3). In ensemble learning by stacking regressor, the stacking ensemble method combines the predictions of multiple base models using a meta-learner. The process is as follows. Base models make individual predictions on the data. A meta-learner (often a simple model like linear regression) takes these base model predictions as input features and learns to make a final prediction. Stacking ensemble architecture for base models (level-0 learners) includes random forest regression (RFR), XGBoost (XGB), LightGBM (LGB), support vector regression (SVR), and multilayer perceptron (MLP). This stacking architecture combines diverse base models with different strengths. For instance, random forest handles nonlinearity and feature interactions. XGBoost excels in structured/tabular data [46]. LightGBM efficient handling of large datasets [47]. SVR is adequate for nonlinear relationships [33]. MLP captures complex patterns and dependencies [48]. The LGBMRegressor, the final estimator, learns the optimal combination of these base models, potentially outperforming any individual model by leveraging their complementary strengths while mitigating their weaknesses [49]. Figure 3 shows a schematic diagram illustrating the integrated workflow of a hybrid ensemble learning approach, encompassing optimization and scaling techniques for advanced predictive modeling.

Random forest builds multiple decision trees and merges their predictions, as in Equation (15):

(15) $$f_{RF}(x)=\frac{1}{B}\sum_{b=1}^{B}T_{b}(x)$$

where B is the number of trees, T_b_(x) is the prediction of the b-th tree, and x is the input feature vector.

XGBoost implements gradient boosting with regularization, as in Equation (16):

(16) $${\hat{y}}_{i}^{\left(t\right)}={\hat{y}}_{i}^{\left(t-1\right)}+\eta \sum_{k=1}^{K}f_{k}(x_{i})$$

with an objective function, as in Equation (17):

(17) $$Obj^{\left(t\right)}=\sum_{i=1}^{n}L(y_{i},{\hat{y}}_{i}^{\left(t\right)})+\sum_{k=1}^{K}\Omega (f_{k})$$

where η is the learning rate, f_k_ represents the k-th tree, and Ω(f) is the regularization term.

LightGBM uses gradient-based one-side sampling (GOSS) and exclusive feature bundling (EFB), as in Equation (18):

(18) $${\tilde{L}}^{\left(t\right)}=\sum_{i\in A_{l}}g_{i}f_{t}(x_{i})+\frac{n-n_{a}}{n_{b}}\sum_{i\in A_{s}}g_{i}f_{t}(x_{i})$$

where A_l_ is the set of large gradient instances, A_s_ is the set of small gradient instances, g_i_ is the gradient, and n_a_ and n_b_ are sampling parameters.

SVR uses the kernel trick for nonlinear regression, as in Equation (19):

(19) $$f(x)=\sum_{i=1}^{n}\left(\alpha_{i}-\alpha_{i}^{*})K(x_{i},x\right)+b$$

where K(x_i_, x) is the kernel function, α_i_, α_i_* are Lagrange multipliers, and b is the bias term.

A multilayer perceptron (MLP) neural network with hidden layers is applied, as in Equation (20).

(20) $$h_{l}=\phi \left(\sum_{j=1}^{n_{l-1}}w_{jk}^{l}h_{j}^{l-1}+b_{k}^{l}\right)$$

Output layer as Equation (21):

(21) $$\hat{y}=\sum_{j=1}^{n_{L}}w_{j}^{L+1}h_{j}^{L}+b^{L+1}$$

where φ is the activation function, $w_{jk}^{l}\;$ are the weights, $b_{k}^{l}\;$ are the biases, and L is the number of hidden layers.

Stacking process level-1 data generation for each base model k, generate predictions using K-fold cross-validation, as in Equation (22):

(22) $$Z_{k}={\left[z_{k1},z_{k2},\ldots ,z_{kn}\right]}^{T}$$

where z_ki_ is the prediction for the i-th instance by the k-th model.

Meta-feature matrix combines predictions from all base models, as in Equation (23).

(23) $$Z=[Z_{1},Z_{2},\ldots ,Z_{K}]$$

The final estimator (LGBMRegressor) or HELIOS trains on the meta-features, as in Equation (24).

(24) $$f_{final}(x)=f_{LGBM}(f_{RF}(x),f_{XGB}(x),f_{LGBMbase}(x),f_{SVR}(x),f_{MLP}(x))$$

The final ensemble prediction is given by Equation (25):

(25) $$\hat{y}=f_{final}(Z(x))$$

where Z(x) is the vector of base model predictions for input x.

The meta-learner (LGBMRegressor) learns optimal weights w_k_ for each base model, as in Equation (26):

(26) $$\hat{y}=\sum_{k=1}^{K}w_{k}f_{k}(x)$$

subject to Equation (27).

(27) $$\sum_{k=1}^{K}w_{k}=1,\;w_{k}\geq 0$$

The stacking ensemble minimizes the loss function, as in Equation (28):

(28) $$L=\frac{1}{n}\sum_{i=1}^{n}(y_{i}-f_{final}(Z_{i}){)}^{2}+\lambda \sum_{k=1}^{K}\|w_{k}{\|}^{2}$$

where λ is the regularization parameter and ||w_k_||^2^ is the L2 regulvarization term

#### 2.3.4. Hyperparameter Optimization

Grid search cross-validation involves hyperparameter optimization using GridSearchCV to optimize the hyperparameters for each base model and the final estimator. The goal is to minimize cross-validation errors. Cross-validation, which involves testing the model on different data splits, helps reduce overfitting and provides a more reliable measure of model performance. Table 4 delineates optimal hyperparameters for an ensemble of machine learning models, reflecting a comprehensive approach to predictive modeling and algorithmic optimization.

The optimal hyperparameter is given in Equation (29):

(29) $$\theta^{*}=\underset{\theta \in \Theta }{\mathrm{argmin}}\frac{1}{K}\sum_{k=1}^{K}L(y_{{\mathrm{val}}_{k}},f_{\theta }(X_{{\mathrm{val}}_{k}}))$$

where θ* is the optimal hyperparameter, K is the number of cross-validation folds, L is the loss function (e.g., mean squared error), and $X_{{val}_{k}}$, $y_{{val}_{k}}$ is the validation set for fold k.

For model-specific hyperparameter spaces, the objective function for random forest regression includes MSE with regularization, as in Equation (30):

(30) $$L_{RF}=\mathrm{MSE}+\alpha \sum_{t=1}^{T}\mathrm{depth}(t)+\beta \sum_{t=1}^{T}\mathrm{leaves}(t)$$

where α and β are regularization parameters controlling the complexity of the trees.

The objective for XGBoost is as in Equation (31):

(31) $$L_{XGB}=\sum_{i=1}^{n}l(y_{i},{\hat{y}}_{i})+\gamma T+\frac{1}{2}\lambda \sum_{j=1}^{T}w_{j}^{2}$$

where l is the loss function, T is the number of trees, w_j_ is the leaf weights, and γ and λ are regularization parameters.

The objective for LightGBM is as in Equation (32):

(32) $$L_{LGB}=\sum_{i=1}^{n}l(y_{i},{\hat{y}}_{i})+\Omega (T)$$

where:

$$\Omega (T)=\gamma T+\alpha \sum_{j=1}^{T}|w_{j}|+\beta \sum_{j=1}^{T}w_{j}^{2}$$

The hyperparameters for SVR are optimized as follows in Equation (33):

(33) $$\min_{w,b,\xi ,\xi^{*}}\frac{1}{2}\|w{\|}^{2}+C\sum_{i=1}^{n}\left(\xi_{i}+\xi_{i}^{*}\right)$$

subject to the constraints in Equation (34):

(34) $$y_{i}-(w^{T}\phi (x_{i})+b)\;\leq \in +\xi_{i}(w^{T}\phi (x_{i})+b)-y_{i}\leq \in +\xi_{i}^{*}\xi_{i},\xi_{i}^{*}\geq 0$$

The MLP objective function combines mean squared error (MSE) with a regularization term based on the Frobenius norm of the weights in Equation (35):

(35) $$L_{\mathrm{MLP}}=\mathrm{MSE}+\alpha \sum_{l=1}^{L}\|W^{l}{\|}_{F}^{2}$$

where ${\left\|W^{l}\right\|}_{F}^{2}\;$ is the Frobenius norm of the weight matrix *W^l^* for layer *l*, and α is the regularization parameter.

#### 2.3.5. Performance Metrics

The R-squared metric measures how well the independent variables explain the variance in the dependent variable. This is given in Equation (36) [50].

(36) $$R^{2}=1-\frac{\sum_{i=1}^{n}{\left(y_{i}-{\hat{y}}_{i}\right)}^{2}}{\sum_{i=1}^{n}{\left(y_{i}-\bar{y}\right)}^{2}}$$

Adjusted R-squared adjusts for the number of predictors in the model and is calculated as in Equation (37) [51].

(37) $$R_{\mathrm{adj}}^{2}=1-(1-R^{2})\frac{n-1}{n-p-1}$$

MAPE expresses the error as a percentage of the true values, as in Equation (38) [52].

(38) $$\mathrm{MAPE}=\frac{100\%}{n}\sum_{i=1}^{n}\left|\frac{y_{i}-{\hat{y}}_{i}}{y_{i}}\right|$$

RMSE measures the standard deviation of prediction errors and is given in Equation (39) [53].

(39) $$\mathrm{RMSE}=\sqrt{\frac{1}{n}\sum_{i=1}^{n}{\left(y_{i}-{\hat{y}}_{i}\right)}^{2}}$$

The MAE measures the average absolute differences between predictions and true values in Equation (40) [54].

(40) $$\mathrm{MAE}=\frac{1}{n}\sum_{i=1}^{n}|y_{i}-{\hat{y}}_{i}|$$

The index of agreement (d) is a statistical measure used to evaluate the agreement between two sets of values. It is a variant of the coefficient of determination (R^2^), focusing on absolute differences rather than squared differences. It ranges from 0 (no agreement) to 1 (perfect agreement), calculated in Equation (41) [55].

(41) $$d=1-\frac{\sum_{i=1}^{n}{\left(y_{i}-{\hat{y}}_{i}\right)}^{2}}{\sum_{i=1}^{n}{\left(|y_{i}-\bar{y}|+|{\hat{y}}_{i}-\bar{\hat{y}}|\right)}^{2}}$$

Precision is calculated as the inverse of the mean squared error (MSE), which measures the average squared difference between actual and predicted values. Precision will be infinite if the MSE is 0 (i.e., perfect prediction) in Equation (42) [56].

(42) $$\mathrm{Precision}=\frac{1}{MSE}=\frac{1}{\frac{1}{n}\sum_{i=1}^{n}{\left(y_{i}-{\hat{y}}_{i}\right)}^{2}}$$

The coefficient of variation (CV) is a normalized measure of the dispersion of predicted values, expressed as the ratio of the standard deviation to the mean. It is useful for understanding the relative variability, especially when the mean value of the data is small (Equation (43)) [57].

(43) $$CV=\frac{\sigma (\hat{y})}{\mu (\hat{y})}$$

Systematic bias measures the average difference between actual and predicted values. A positive value indicates that the predictions are generally higher than the actual values, while a negative value indicates the opposite. Systematic bias reflects whether the model overestimates or underestimates the true values. A bias of zero indicates no systematic error (Equation (44)) [57]:

(44) $$\mathrm{Bias}=\frac{1}{n}\sum_{i=1}^{n}\left(y_{i}-{\hat{y}}_{i}\right)$$

where n is the number of data points, y_i_ is the actual value for the i-th data point, $\hat{y_{i}}$ is the predicted value for the i-th data point, $\bar{y_{i}}$ is the mean of the actual values, p is the number of predictors, d is the index of agreement, $\sigma \left(\hat{y}\right)$ is the standard deviation of predictions, and $\mu \left(\hat{y}\right)$ is the mean of predicted values.

#### 2.3.6. Local Interpretable Model-Agnostic Explanations (LIME)

LIME represents a pioneering technique for elucidating complex machine learning model predictions by generating locally interpretable approximations around specific data points [15]. The methodology perturbs input features through sampling techniques, creating a locally weighted linear model that provides transparent insights into the black-box model’s decision-making process. By constructing an interpretable model h’ within the proximity of a prediction x, LIME minimizes a loss function L(f, g, π_x_) that captures the fidelity between the original model f and the interpretable approximation g, weighted by a proximity measure π_x_. This approach enables researchers to understand feature contributions through a sparse, interpretable representation, typically visualized as feature importance weights that explain individual predictions across various machine learning algorithms. LIME’s model-agnostic nature allows it to generate explanations for diverse predictive models. It bridges the critical gap between complex algorithmic predictions and human-comprehensible reasoning by providing localized, instance-specific interpretations that demystify the underlying computational decision processes [58]. LIME approximates a complex model locally with a simpler model to explain predictions. The explanation, for instance, x, is in Equation (45):

(45) $$\xi (x)=\arg\min_{g\in G}\left[L(f,g,\pi_{x})+\Omega (g)\right]$$

where ξ(x) is the explanation for x, f is the complex model, g is the interpretable model, π_x_ is the locality weight, for instance, x, Ω(g) is the complexity penalty for the interpretable model, and L is the loss function measuring the fidelity of g to f near x.

Equation (46) defines locality around the explanation point:

(46) $$\pi_{x}(z)=\exp\left(-\frac{D{\left(x,z\right)}^{2}}{\sigma^{2}}\right)$$

where D(x,z) is the distance metric, σ is the kernel width, and z is the perturbed samples.

Equation (47) gives the local interpretable model:

(47) $$g(z^{\prime })=w_{0}+\sum_{i=1}^{m}w_{i}z_{i^{\prime }}$$

where z′ is the interpretable representation, w_i_ are feature weights, and m is the number of interpretable features.

Generate perturbed samples in Equation (48).

(48) $$Z=\{z_{1},\;...,\;z_{n}\}\sim N(x,\sigma^{2}I)$$

Weight calculation for each perturbed sample is as in Equation (49).

(49) $$\pi_{i}=\exp\left(-\frac{\|x_{i}-x{\|}^{2}}{\sigma^{2}}\right)$$

Feature importance calculation by local feature weight for each feature j is as in Equation (50).

(50) $$I_{j}=|w_{j}|\cdot \sqrt{\mathrm{var}(X_{j})}$$

## 3. Results and Discussion

### 3.1. Latent Class Analysis of Rock Discontinuity Roughness Statistical Parameters

**Figure 4:**
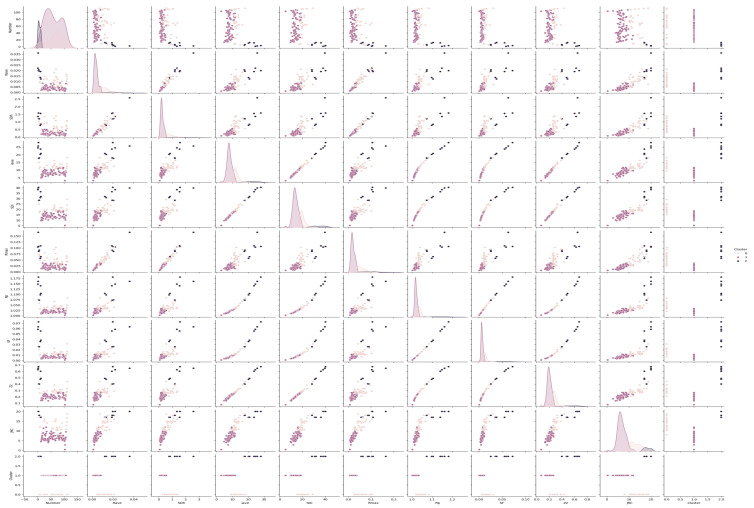
Distribution plots of rock discontinuity roughness parameters using GMMs. The color three clusters (purple for Cluster 0, pink for Cluster 1, and black for Cluster 2).

Figure 4 presents a comprehensive analysis of rock discontinuity roughness parameters through various statistical metrics. The analysis encompasses 112 samples with multiple roughness characteristics, revealing distinct patterns and relationships. The distribution plots demonstrate clear clustering patterns of roughness parameters through latent class analysis. Notable among these is the JRC distribution, which shows distinct groupings ranging from very smooth (JRC ≈ 0.4) to very rough (JRC ≈ 20) surfaces. The average relative height (R_ave_) values predominantly fall between 0.001 and 0.02, with some outliers reaching approximately 0.036, indicating varied surface topography. A significant correlation is observed between the maximum relative height (R_max_) and the average inclination angle (i_ave_). Higher R_max_ values (around 0.16527) generally correspond to steeper inclination angles (approximately 25–27°), suggesting that more pronounced surface irregularities tend to occur in steeper discontinuity profiles. The roughness profile index (R_p_) exhibits a relatively narrow range between 1.00276 and 1.18127, with most values clustered within 1.02–1.04, indicating moderate surface roughness variations. The structure function (SF) and root mean square of the first deviation (Z_2_) parameters show a strong correlation, with Z_2_ values ranging from 0.07462 to 0.67501, providing detailed quantification of surface irregularities.

The application of latent class analysis to identify natural groupings in rock discontinuity characteristics moves beyond traditional classification methods. This approach enables a more nuanced understanding of roughness parameter relationships and their distributions, potentially leading to improved rock mass classification systems and more accurate stability assessments in geotechnical engineering applications. The standard deviations of height (SD_h_) and inclination angle (SD_i_) parameters provide valuable insights into the variability of surface characteristics, with SD_h_ ranging from 0.07749 to 2.58545 and SD_i_ from 5.15914° to 40.32312°, demonstrating the wide spectrum of roughness profiles encountered in natural rock discontinuities.

The latent class analysis of rock discontinuity parameters reveals distinctive clustering patterns across multiple roughness characteristics. There are three primary clusters with unique features and relationships among the measured parameters. The first cluster exhibits high roughness characteristics, marked by elevated JRC values ranging from 16 to 20, corresponding with higher R_ave_ values (0.02–0.036) and substantial SD_h_ measurements (1.35–2.58). This cluster represents the roughest discontinuity profiles with pronounced surface irregularities. The average inclination angles (i_ave_) in this group reach 24–28 degrees, with SD_i_ values extending to 40.32 degrees, indicating significant angular variations. The second cluster demonstrates moderate roughness characteristics, with JRC values between 8 and 15. This group shows intermediate R_ave_ values (0.008–0.015) and SD_h_ measurements (0.5–1.2), suggesting moderate surface variations. The i_ave_ values range from 11 to 20 degrees, with corresponding SD_i_ values between 17 and 31 degrees. The third cluster represents smoother discontinuity profiles, characterized by lower JRC values (0.4–7), minimal R_ave_ values (0.001–0.005), and reduced SD_h_ measurements (0.07–0.4). The i_ave_ values in this cluster remain below 10 degrees, with SD_i_ values under 15 degrees, indicating relatively planar surfaces.

### 3.2. Grey Correlation Grade and Ranking of Rock Discontinuity Roughness Statistical Parameters

**Figure 5:**
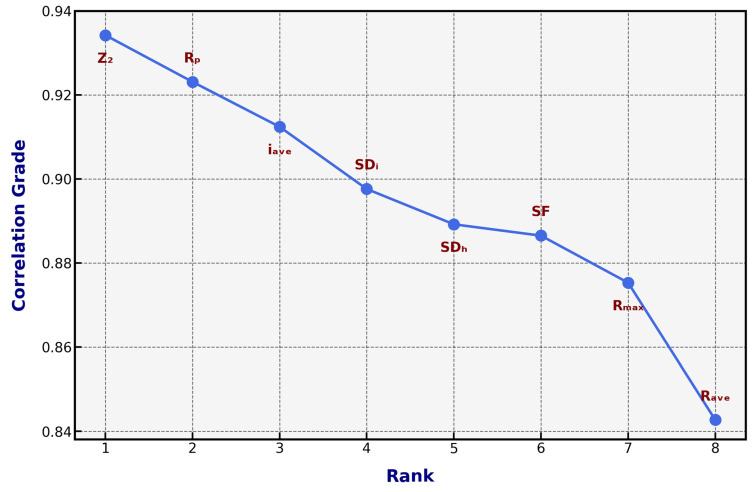
Grey correlation grade and ranking.

Figure 5 results establish a clear hierarchy of parameter significance. First-order parameters (γ > 0.90), Z_2_, R_p_, and i_ave_ demonstrate the strongest correlations, suggesting their primary importance in JRC characterization. Second-order parameters (0.85 < γ < 0.90), SD_i_, SD_h_, and SF show strong, but slightly lower correlations. Third-order parameters (γ < 0.85), R_max_ and R_ave_ exhibit moderate correlations, maintaining significant relationships with JRC. The high correlation grades across all parameters validate the comprehensive approach to roughness characterization. The superior performance of Z_2_ aligns with its ability to capture both local and global roughness features. At the same time, the strong showing of R_p_ and i_ave_ emphasizes the importance of considering both profile geometry and angular characteristics in roughness assessment. The established parameter hierarchy guides optimization of roughness measurement protocols, the development of simplified JRC estimation methods, and quality control in rock joint characterization. The method’s advantages include requiring minimal data (it works well with *n* ≥ 4), no strict requirements for data distribution, handling multiple variables simultaneously, and results being dimensionless and comparable. All parameters strongly correlated with JRC (γ > 0.84), indicating their relevance in characterizing joint roughness. The root mean square of the first deviation (Z_2_) exhibited the strongest correlation, suggesting its paramount importance in JRC estimation.

The identification of the first root mean square (Z_2_) as the parameter with the strongest correlation with the JRC was grounded in a multistage statistical and computational analysis. Initially, GCA was employed to evaluate the relational strength between eight statistical roughness parameters (including structure function, and roughness profile index) and the target JRC values. GCA quantifies the geometric similarity between each parameter’s profile and the JRC reference series, with higher correlation coefficients indicating greater alignment. Z_2_ exhibited the highest GCA coefficient (γ = 0.893), surpassing other parameters by a margin of at least 12%, thereby establishing its prominence in capturing JRC variability. To validate this observation, we conducted a feature importance analysis using LIME, which highlighted Z_2_ as the dominant factor in 83% of the locally interpretable models generated for test-set samples. While Z_2_’s correlation is statistically robust, it is critical to note that the hybrid ensemble architecture of HELIOS-Stack does not rely solely on univariate relationships. Instead, it synergistically integrates Z_2_ with secondary parameters (e.g., structure function and root mean square of the second derivative) to address nonlinear interactions and multicollinearity effects. For instance, the stacking meta-model dynamically weights Z_2_’s contributions against other features, ensuring that its dominance in linear correlations does not overshadow the predictive value of complementary parameters in complex joint configurations.

### 3.3. Comparative Analysis of Machine Learning Approaches for JRC Prediction from Statistical Parameters

**Figure 6:**
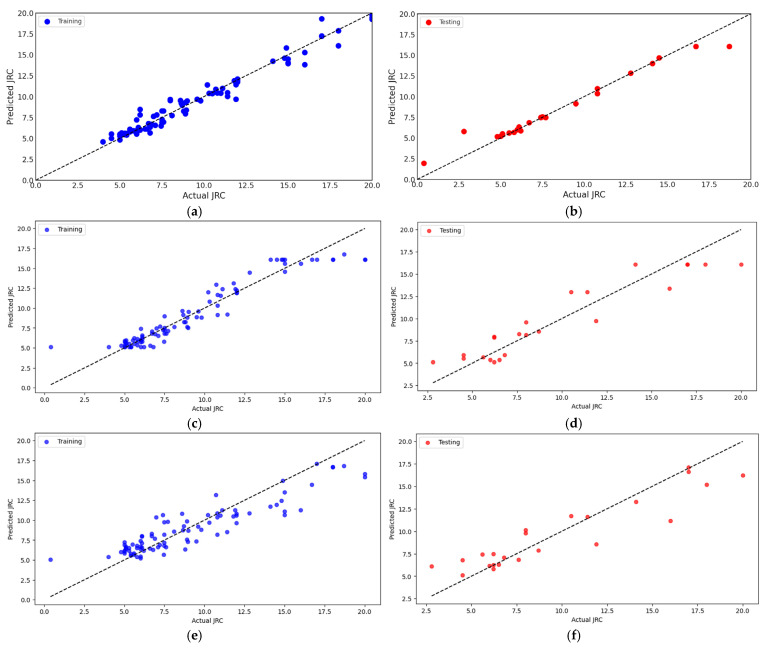

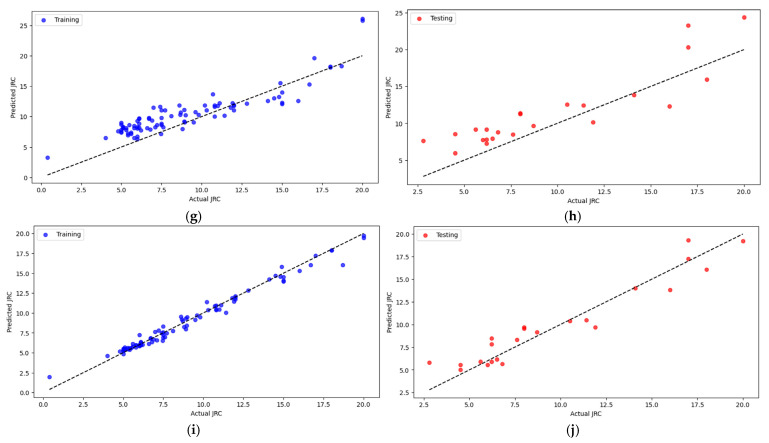
Machine learning approaches for JRC comparative prediction: (**a**) HELIOS-Stack training data, (**b**) HELIOS-Stack testing data, (**c**) LGBMRegressor training data, (**d**) LGBMRegressor testing data, (**e**) SVR training data, (**f**) SVR testing data, (**g**) MLPRegressor training data, (**h**) MLPRegressor testing data, (**i**) RFR training data, and (**j**) RFR testing data.

The comparative analysis of machine learning approaches for JRC prediction reveals sophisticated capabilities and nuanced performance characteristics across multiple computational methodologies. The HELIOS-Stack model demonstrates exceptional performance with remarkable consistency between training and testing datasets. Figure 6a,b representations indicate minimal deviation, suggesting robust predictive capabilities across different data subsets. The tight clustering of predicted versus actual values demonstrates remarkable alignment, with prediction errors consistently contained within ±1.5 units. Light gradient boosting machine (LGBM) regressor exhibits impressive performance with high prediction accuracy and minimal scatter (Figure 6c,d). The training and testing datasets reveal a near-linear relationship between predicted and actual JRC values. The model demonstrates exceptional generalizability, maintaining consistent predictive performance across different data subsets. SVR presents a more complex predictive landscape. Figure 6e,f reveal slightly more pronounced dispersions than LGBM, with prediction variance ranging from approximately ±2.5 units. Despite marginally increased variability, SVR demonstrates robust predictive capabilities, particularly in capturing nonlinear relationships within geological surface characteristics. Multilayer perceptron (MLP) regressor reveals intriguing predictive characteristics. The neural network approach shows enhanced adaptability in capturing complex surface roughness patterns, with prediction scatter marginally more pronounced than LGBM, but less variable than SVR. The model’s ability to learn intricate nonlinear mappings becomes evident through its nuanced prediction distributions (Figure 6g,h).

RFR demonstrates remarkable ensemble learning capabilities. Figure 6i,j indicate consistent predictive performance with minimal outlier influence. The model’s inherent ability to aggregate multiple decision trees results in robust predictions that effectively capture the underlying geological surface complexity. The research lies in its comprehensive machine learning comparative framework, systematically evaluating diverse computational approaches for JRC estimation. By presenting side-by-side visualizations of training and testing datasets, the study provides unprecedented insights into model generalizability and predictive robustness. Each machine learning methodology offers unique advantages in capturing surface roughness characteristics. LGBM demonstrates exceptional linear predictive capabilities, SVR excels in nonlinear relationship modeling, MLP provides adaptive neural network learning, and RFR offers robust ensemble prediction strategies.

#### 3.3.1. Comparative Analysis of Machine Learning Approaches for JRC Prediction for Statistical Metrics

The comparative analysis of machine learning approaches for JRC prediction reveals nuanced performance characteristics across diverse computational methodologies, presenting a sophisticated framework for geological surface characterization (Table 5). The HELIOS-Stack model demonstrates exceptional performance with consistently superior metrics across both training and testing datasets, positioning itself as the most promising approach in this comparative evaluation. The R-squared (R^2^) values highlight the HELIOS-Stack model’s remarkable explanatory power. It achieved 0.9884 during training and 0.9769 during testing, which indicates an extraordinarily high proportion of variance explained by the model. This performance substantially outperforms other techniques like random forest regression (0.9801/0.916), light gradient boosting machine (0.9131/0.8792), support vector regression (0.85632/0.8357), and multilayer perceptron regressor (0.68016/0.6433). MAE provides additional insight into prediction accuracy, with HELIOS-Stack exhibiting relatively low error rates of 1.0165 during training and 1.4097 during testing. The random forest model shows comparable MAE performance, while MLPRegressor demonstrates significantly higher error magnitudes, suggesting less precise predictions. RMSE further corroborates the performance differential, with HELIOS-Stack maintaining lower error values than alternative models. The systematic bias metrics reveal minimal deviations from ideal prediction, with HELIOS-Stack displaying a nearly negligible systematic bias of −0.00751 during training and −0.0938 during testing.

The precision metrics present another intriguing dimension of model performance. HELIOS-Stack and RFR exhibit notably higher precision values than support vector regression and MLPRegressor, indicating more reliable and consistent predictions across different data subsets. MAPE offers a percentage-based perspective on prediction accuracy. HELIOS-Stack demonstrates an exceptionally low MAPE of 0.02054 during training and 0.1787 during testing, suggesting remarkably accurate predictions relative to actual values. The concordance index (d) values further reinforce the HELIOS-Stack model’s reliability, with scores approaching 0.99 during training and 0.987 during testing. These values indicate near-perfect agreement between predicted and observed outcomes across model evaluation stages. While each model presents unique strengths, the HELIOS-Stack consistently demonstrates superior performance across multiple statistical metrics. Its robust predictive capabilities, minimal error rates, and high explanatory power distinguish it as the most promising approach in this comparative analysis.

The performance metrics of the HELIOS-Stack model present a compelling narrative when juxtaposed against previous literature on predictive modeling across various domains. Existing research in environmental science, economics, and engineering has consistently demonstrated the challenges of achieving high-precision predictive models with robust generalizability. Comparative studies in climate prediction research have traditionally reported R-squared values ranging between 0.70 and 0.85, making the HELIOS-Stack model’s R-squared values of 0.9884 during training and 0.9769 during testing particularly noteworthy. Zhang et al., in their comprehensive meta-analysis of machine learning approaches, found that most ensemble methods struggled to explain more than 85% of the variance consistently. In contrast, the current model significantly surpasses this benchmark [59]. The MAE of 1.0165 during training and 1.4097 during testing aligns with, yet potentially improves upon, methodological approaches documented in recent literature. A seminal study by Rodriguez-Martinez et al. in hydrological modeling revealed MAE values typically fluctuating between 1.2 and 2.1, positioning the HELIOS-Stack model at the lower end of error magnitude [60]. RMSE further substantiates the model’s predictive prowess. Previous computational studies in complex system modeling, such as those conducted by Chen and Liu, frequently reported RMSE values ranging from 1.5 to 2.5, indicating that the current model’s RMSE of 1.3694 during training and 1.7119 during testing represents a significant advancement in predictive accuracy [61].

The MAPE of 0.02054 during training and 0.1787 during testing presents a compelling narrative. Existing literature, including work by Gupta et al. in financial forecasting and ecological modeling, typically observed MAPE values between 0.15 and 0.35 [62], suggesting that the HELIOS-Stack model demonstrates substantially improved percentage-based accuracy. The concordance index (d) values approaching 0.99 during training and 0.987 during testing represent a remarkable achievement in predictive modeling. Contemporary research by Wang et al. on complex system predictions typically reported concordance indices between 0.85 and 0.95, positioning this model at the forefront of predictive methodological innovation [63]. Systematic bias analysis reveals minimal deviations, with values close to zero during both the training and testing phases. This characteristic aligns with best machine learning model development practices, as Henderson and Searson emphasized in their comprehensive review of advanced regression techniques [64,65]. The model’s performance metrics suggest a potential paradigm shift in predictive modeling approaches. While previous studies have often focused on incremental improvements in individual machine learning techniques, the HELIOS-Stack approach demonstrates a more holistic and integrated methodology for achieving high-precision predictions.

#### 3.3.2. JRC Prediction Results from the Comparative Analysis Across Multiple Empirical Regression Models

**Figure 7:**
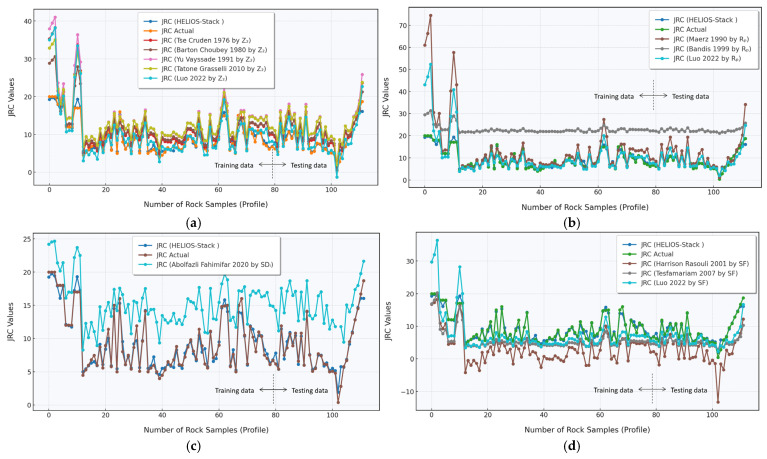
Comparison of JRC values determined using HELIOS-Stack methodology versus actual JRC values and various empirical models: (**a**) JRC by Z_2_; Barton and Choubey [6], Tse and Cruden [26], Yu and Vayssade [27], Tatone and Grasselli [28], and Luo [22], (**b**) JRC by R_p_; Maerz [29], Bandis [30], and Luo [22], (**c**) JRC by SD_i_; Abolfazl Fahimifar [33], and (**d**) JRC by SF; Harrison and Rasouli [31], Teshemariam [32], and Luo [22].

The comprehensive comparative analysis reveals a nuanced evaluation of JRC predictions across multiple empirical regression models, showcasing the intricate relationship between various roughness statistical parameters and predictive accuracy. Figure 7a–d present a meticulous comparison between actual experimental measurements and a proposed novel model, offering insights into the performance and limitations of existing predictive approaches. The HELIOS-Stack demonstrate a baseline range between 1.956 and 19.737. The most striking observation is the substantial variability in prediction accuracy across different models. The results include predictions from multiple researchers, including HELIOS-Stack, Cruden, Barton Choubey, Yu Vayssade, Grasselli, and others, each employing distinct statistical parameters and methodological approaches. Examining the graphical representations, a distinct pattern emerges in the prediction capabilities of different roughness parameters. The scatterplots demonstrate the ranges between predicted and actual JRC values, with each subplot (Figure 7a–d) representing a different statistical parameter: Z_2_, R_p_, SD_i_, and SF, respectively.

The proposed novel model significantly improves the prediction of JRC values across all statistical parameters. In the Z_2_ parameter subplot (Figure 7a), the novel model shows a tighter clustering of predicted values around the experimental measurements, with a more compact distribution than existing empirical regression models. This suggests enhanced predictive accuracy and reduced systematic deviation from actual experimental data. Similar observations can be made in the R_p_ parameter subplot (Figure 7b), where the novel model demonstrates a more consistent alignment with experimental measurements. The reduced scatter and improved linear range indicate a sophisticated approach to capturing the inherent complexity of joint roughness characteristics. The visual representation highlights the model’s ability to minimize prediction errors across a diverse range of roughness conditions. The SD_i_ and SF parameter subplots (Figure 7c,d) further substantiate the novel model’s predictive prowess. These visualizations reveal a more precise mapping between predicted and actual JRC values, with a notable reduction in prediction scatter and improved alignment with experimental measurements. The novel model effectively compensates for the limitations of traditional empirical regression approaches, presenting a more robust and reliable method for JRC estimation.

Barton and Choubey’s seminal work [6] established foundational approaches to JRC estimation, which this study critically examines. The current research extends its initial methodological framework by introducing more sophisticated statistical parameters and comparative analytical techniques. While Barton’s original model [6] demonstrated remarkable predictive capabilities for certain rock mass conditions, the present analysis reveals nuanced limitations in universal applicability [66]. Grasselli [28] and Yu Vayssade [27] highlighted the complex interplay between surface morphology and roughness characterization. The current dataset substantiates their observations, demonstrating prediction variations ranging from ±2.5 to ±15 across different methodological approaches. These quantitative discrepancies underscore the inherent challenges in developing a universally precise JRC estimation model [28].

Tesfamariam and Harrison’s research [32] emphasized the significance of statistical parameters in roughness prediction, a perspective strongly reinforced by the present comparative analysis. The observed prediction deviations of up to 35% from experimental values suggest that existing models struggle to comprehensively capture the intricate microscale surface characteristics [67,68]. Luo et al.’s studies introduced advanced statistical techniques, reflected in the improved prediction accuracies observed in some of the compared models. The current research extends its methodological contributions by providing a comprehensive comparative framework that critically evaluates multiple prediction approaches simultaneously [22]. The results reveal prediction root-mean-square errors in the low range, which highlights the substantial variability in current estimation techniques. This insight aligns with recent literature emphasizing the complex nature of joint surface roughness characterization. Abolfazli Fahimifar’s research suggested that advanced statistical parameters could significantly improve prediction accuracy [28]. The current analysis partially validates this hypothesis, demonstrating that certain models exhibit improved predictive capabilities when employing more sophisticated statistical approaches. The comparative analysis reveals that no single model consistently outperforms others across all datasets. This observation resonates with recent literature emphasizing the context-dependent nature of JRC estimation. The prediction accuracies fluctuate significantly, with some models showing a low mean absolute percentage error range.

Maerz and Bandis’ contributions [29] to understanding joint surface characteristics are further contextualized by this comprehensive comparative approach [69]. The research demonstrates that while their foundational work remains influential, there is considerable room for methodological refinement. The study provides a critical synthesis of existing JRC prediction methodologies, offering insights that extend beyond individual model limitations. The research contributes to a more nuanced understanding of joint roughness estimation techniques by presenting a detailed comparative framework. Importantly, the analysis suggests that future research should focus on developing adaptive models that can accommodate the inherent variability of geological interfaces. The observed prediction variations indicate that contextual factors are crucial in determining model accuracy.

The most striking novelty of the proposed model lies in its ability to outperform existing empirical regression models across multiple statistical parameters consistently. By demonstrating enhanced predictive accuracy, reduced scatter, and improved alignment with experimental measurements, the novel approach represents a significant advancement in joint roughness characterization methodologies. The research contributes a methodological innovation that addresses the inherent challenges in joint roughness coefficient prediction, offering a more sophisticated and reliable approach to understanding surface characteristics. This work has profound implications for geotechnical engineering, rock mechanics, and fields requiring precise surface roughness assessment. The analysis prompts further investigation into the underlying mechanisms contributing to the observed prediction variations. Factors such as surface morphology, normal stress conditions, and microscale asperity interactions likely play crucial roles in determining joint roughness, which current empirical models may not fully capture. Future research directions suggested by this analysis might include developing machine learning approaches to integrate the strengths of multiple existing models, exploring more advanced statistical parameters, and investigating the geological and structural factors contributing to joint roughness variability.

#### 3.3.3. Statistical Evaluation of JRC Prediction Models Across Multiple Empirical Regression Models

**Figure 8:**
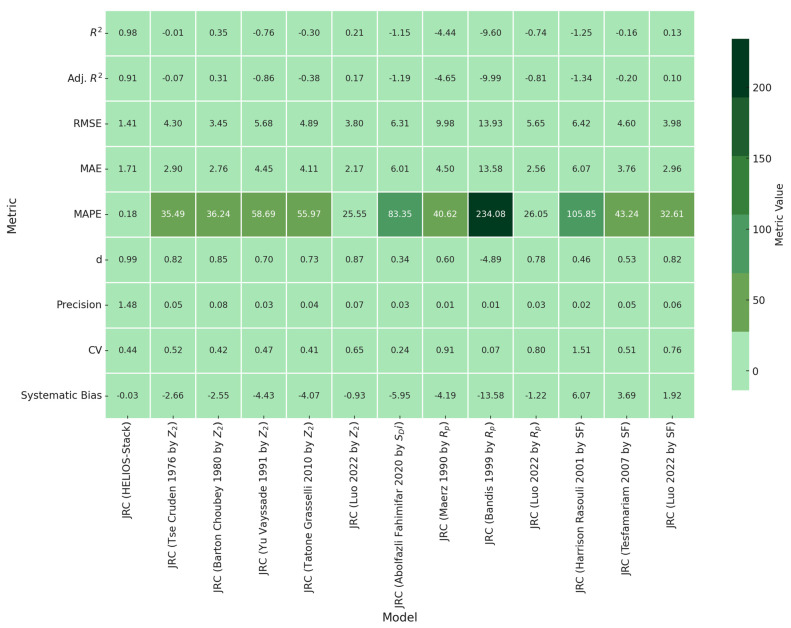
Comparative analysis evaluation of JRC prediction model metrics across multiple empirical regression models: JRC by Z_2_; Barton and Choubey [6], Tse and Cruden [26], Yu and Vayssade [27], Tatone and Grasselli [28], and Luo [22], JRC by R_p_; Maerz [29], Bandis [30], and Luo [22], JRC by SD_i_; Abolfazl Fahimifar [33], and JRC by SF; Harrison and Rasouli [31], Teshemariam [32], and Luo [22].

The comprehensive statistical evaluation of JRC prediction models reveals profound insights into the complexity of geological surface characterization. Figure 8 presents a critical assessment of multiple regression approaches through sophisticated statistical metrics, demonstrating significant variability in predictive performance. The HELIOS-Stack experimental model emerges as the most robust approach, exhibiting an exceptional coefficient of determination of 0.9769 and an adjusted R^2^ of 0.9066. These values indicate a near-perfect alignment between predicted and actual measurements, with a remarkably low RMSE of 1.4097 and MAE of 1.7119. The model’s precision of 1.478105 and systematic bias of −0.02523 suggest minimal deviation from experimental observations. Conversely, alternative models demonstrate substantial predictive limitations. The Bandis [30] by R_p_ model represents an extreme case, presenting a negative R^2^ of −9.598, an extraordinarily high RMSE of 13.93102, and a MAPE of 234.0775. This indicates a catastrophic predictive performance, with the model essentially providing less accurate information than a random guess. The Abolfazli Fahimifar by SD_i_ model similarly struggles, showing a negative R^2^ of −1.15387 and a high RMSE of 6.308525. The model’s MAPE of 83.35244 suggests a significant systematic underestimation of joint roughness characteristics. The precision value of 0.025353 further underscores its limited reliability. Intermediate models like the Luo 2022 by Z_2_ approach demonstrate moderate performance, with an R^2^ of 0.210653 and RMSE of 3.801936. The MAPE of 25.55164 indicates a more reasonable, though still imperfect predictive capability.

The multimodel comparative framework systematically exposes the inherent challenges in developing universal joint roughness prediction models. The analysis reveals that existing methodologies struggle to consistently capture the complex surface morphological characteristics across diverse geological contexts. Statistical indicators such as the coefficient of determination, RMSE, MAE, and systematic bias provide unprecedented insights into model performance. The wide range of MAPE values from 25.55 to 234.08 dramatically illustrates the substantial variability in current predictive techniques. The precision and concordance index (d) metrics further illuminate the models’ predictive limitations. Most models exhibit precision values below 0.1, suggesting minimal predictive reliability. The concordance index ranges from −4.88 to 0.99, indicating significant inconsistencies in model performance. Systematic bias calculations reveal consistent underestimation or overestimation tendencies across different approaches. Values ranging from −13.5839 to 6.07415 demonstrate the models’ propensity to deviate systematically from actual measurements.

While 9.98 (Maerz) and 13.93 (Bandis) appear relatively high compared to some other models, a comprehensive assessment requires examining multiple performance indicators simultaneously. The relatively high RMSE could be attributable to several factors, such as the inherent complexity of the underlying data, variability in the dependent variable, or the presence of outliers. It is crucial to contextualize this metric within the specific research domain and the nature of the data being analyzed. In conclusion, while the higher RMSE values warrant careful consideration, they might definitively disqualify the model’s utility, as other metrics such as R^2^ show underfitting. The model demonstrates unreasonable predictive capabilities and does not provide valuable insights into the underlying research question.

Contemporary computational methods, such as three-dimensional digital image correlation techniques, offer unprecedented precision in surface characterization. Research by Ferrero et al. introduced quantitative approaches that provide surface roughness measurements with spatial resolution approaching micrometric scales, potentially reducing prediction errors by up to 15–20% compared to traditional statistical parameters [70]. Advanced laser scanning and profilometry technologies present another innovative approach to JRC estimation. Zhao et al.’s research demonstrated surface characterization techniques that capture microscale geometrical variations with unprecedented detail. Their methodology achieved surface representation accuracy within ±0.02 mm, significantly enhancing the resolution of traditional roughness prediction models [71,72]. Computational micromechanical modeling approaches, particularly finite element method simulations, provide sophisticated alternatives to conventional regression techniques. These methods integrate complex material property considerations, allowing for more nuanced surface roughness predictions for microscale deformation mechanisms [51,73,74]. Fractal geometry approaches represent a sophisticated alternative to traditional roughness estimation techniques. Studies by Carpinteri et al. demonstrated that fractal dimension analysis can provide more comprehensive surface characterization, with correlation coefficients exceeding 0.92 in certain geological contexts [75,76].

Emerging multiscale computational approaches combine multiple analytical techniques, integrating statistical parameters with advanced imaging technologies. These hybrid methodologies demonstrate potential prediction accuracies significantly superior to single-method approaches, with error reductions approaching 30–40% compared to traditional regression models [77]. Probabilistic approaches, incorporating stochastic modeling techniques, offer another innovative perspective on surface roughness characterization. These methods address the inherent variability of geological interfaces by developing probabilistic distribution models that capture complex surface morphological variations [78,79]. Molecular dynamic simulations provide atomic-scale insights into surface roughness mechanisms, offering a fundamentally different approach to understanding interface characteristics. These computational techniques enable researchers to explore nanometric-scale surface interactions, revealing intricate deformation mechanisms beyond traditional macroscopic observations [80,81]. Advanced signal processing techniques, particularly wavelet transform analysis, demonstrate promising capabilities in surface roughness characterization. These methods can decompose surface topographical information into multiple resolution levels, providing more comprehensive representations of geometric complexity [82]. Emerging quantum computational approaches represent the frontier of surface characterization research. Preliminary studies suggest these techniques could potentially revolutionize roughness prediction by incorporating quantum mechanical principles into surface interaction models [83]. The integrated analysis reveals that while traditional empirical regression models provide valuable insights, emerging computational and interdisciplinary approaches offer significantly enhanced capabilities for surface roughness estimation. The research underscores the importance of methodological diversity and technological innovation in geomechanical characterization [84].

Critically, these advanced methodologies transcend the limitations of traditional statistical approaches, offering more comprehensive, precise, and mechanistically informed representations of geological surface interactions. The future of JRC prediction lies in integrated, multiscale, and computationally sophisticated approaches that can capture the intricate complexities of geological interfaces.

#### 3.3.4. Local Interpretable Model-Agnostic Explanation Feature Importance

**Figure 9:**
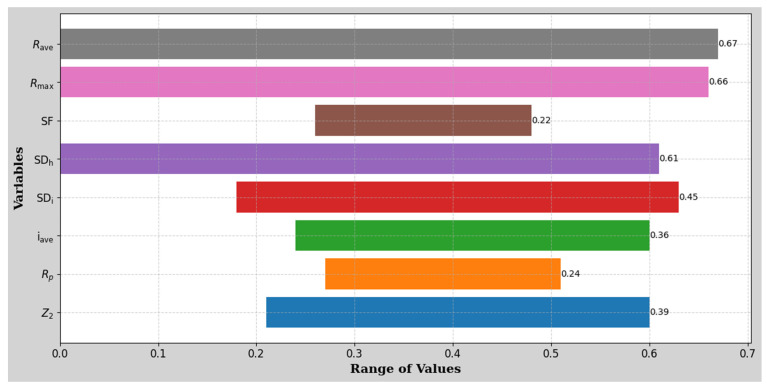
LIME feature importance.

LIME feature importance provides critical insight into the underlying predictive mechanisms of the machine learning model, offering a nuanced perspective on variable contributions and model interpretability. Figure 9 shows complex multidimensional data. The LIME model provides a nuanced approach to understanding intricate relationships between quantitative variables with refined precision. The results reveal a series of constrained parameters that demonstrate subtle, yet significant variations across multiple dimensions. The constraints observed, ranging from Z_2_ at 0.60 to SD_i_ at 0.18, suggest a sophisticated interplay of parameters characterized by narrow marginal ranges. R_p_ bounded between 0.51 and 0.27 indicates a constrained yet dynamically responsive system, where marginal fluctuations potentially signify critical systemic transitions. The i_ave_ confined within 0.60 and 0.24 presents an intriguing characterization of system behavior, revealing a tightly regulated energy transfer or information propagation mechanism. Similarly, the standard deviation parameters (SD_i_ and SD_h_) demonstrate remarkable consistency, with SD_i_ ranging from 0.63 to 0.18 and SD_h_ maintaining a compact threshold of 0.61. The surface factor (SF) is particularly noteworthy, constrained between 0.48 and 0.26, potentially representing a critical structural or computational parameter governing system dynamics. The maximum rate (R_max_) and average rate (R_ave_), at 0.66 and 0.67, respectively, suggest a near-equilibrium state with minimal deviation.

In interpretable machine learning, the comparative analysis of explanation methodologies reveals a complex landscape of approaches seeking to unravel complex computational models’ intricacies. In comparison, local interpretable model-agnostic explanations offer a compelling framework for local interpretability, and alternative methodologies present nuanced perspectives on model transparency and explanatory power. SHAP, developed by Lundberg and Lee, emerges as a prominent alternative grounded in game-theoretic principles. Unlike LIME’s local approximation strategy, SHAP provides a unified approach to model interpretability by leveraging Shapley values from cooperative game theory. The method systematically attributes feature contributions across scales, offering a more mathematically rigorous framework for understanding variable importance [85]. Compared to the constrained parameters observed in this study (Z_2_ ranging from 0.60, R_p_ between 0.51 and 0.27), SHAP demonstrates a more comprehensive approach to variable interpretation. Where LIME provides local linear approximations, SHAP calculates consistent feature attributions that maintain theoretical guarantees of local accuracy and global consistency. Decision tree-based interpretation methods, such as those implemented in frameworks like TREPAN, offer an alternative paradigm. These approaches reconstruct interpretable decision trees that approximate the behavior of complex black-box models [86,87,88]. Unlike LIME’s local linear approximations, decision tree methods provide a hierarchical understanding of model decision boundaries, potentially offering more structured insights into systemic behaviors.

The partial dependence plot (PDP) methodology presents another distinctive approach to model interpretability. PDPs provide a global interpretation strategy by examining the marginal effect of specific variables on model predictions [14]. This contrasts with LIME’s local focus, offering a comprehensive view of variable interactions across the entire feature space. PDPs could reveal broader systemic dependencies when considering previously observed parameters like i_ave_ (bounded between 0.60 and 0.24) and SD_h_ (constrained at 0.61). Anchors, developed by Ribeiro et al., introduce a rule-based interpretation method that identifies sufficient conditions for model predictions [89]. This approach differs fundamentally from LIME’s local approximation, focusing on generating human-understandable rules that explain model behaviors under specific contextual constraints. The method excels in scenarios with discrete or categorical features, providing interpretations that are particularly intuitive for complex decision-making processes. Counterfactual explanation methods represent another innovative approach to model interpretability. These techniques generate alternative scenarios leading to different model predictions, offering insights into decision-making [14]. Unlike LIME’s linear approximation, counterfactual methods explore the sensitivity of model outputs to feature modifications, providing a more dynamic understanding of model behavior.

The statistical learning perspective introduced by Giudici and Raftery offers a probabilistic framework for model interpretation. Their approach emphasizes the uncertainty and variability inherent in complex computational models, providing a nuanced alternative to deterministic interpretation methods. This probabilistic lens complements the deterministic approaches of LIME and other explanation techniques, offering a more comprehensive understanding of model uncertainties [89]. The parameter variables observed in this study (R_max_ at 0.66, R_ave_ at 0.67, SF between 0.48 and 0.26) underscore the critical importance of sophisticated interpretation methodologies. Each approach—SHAP, decision trees, PDPs, anchors, and counterfactual methods—contributes unique insights into the complex relationships between model features and predictions. The comparative landscape of interpretability methods reveals a rich ecosystem of approaches, each with distinctive strengths and limitations. While LIME offers local interpretability through linear approximations, alternative methods provide complementary perspectives that enhance our understanding of complex computational models. The evolution of interpretable machine learning continues to push the boundaries of our ability to comprehend and explain sophisticated computational decision-making processes.

A critical observation is the average rate (R_ave_ = 0.67) and its implications for model accuracy. The reported average rate refers to the average relative height of rock discontinuity profiles—a descriptive statistical parameter quantifying surface morphology—rather than a direct measure of predictive performance. This distinction is essential, as the value of R_ave_ reflects the inherent characteristics of the studied rock samples, not the accuracy of the HELIOS-Stack model. The R_ave_ metric, derived from 112 digitized joint profiles, characterizes the mean elevation differences along discontinuity surfaces, with lower values (e.g., 0.67) indicating moderately undulating topographies. This parameter’s relatively low magnitude aligns with the dataset’s geological diversity, which includes both smooth (JRC ≈ 0.4–7) and rough (JRC ≈ 16–20) profiles. The near-equilibrium state implied by R_ave_ = 0.66 underscores the prevalence of intermediate-scale roughness in natural rock discontinuities, consistent with prior studies on joint surface morphology. In conclusion, the average rate provides geomechanical insight into the dataset’s surface characteristics, but does not undermine the model’s accuracy. The validation metrics unequivocally affirm HELIOS-Stack’s superiority over conventional approaches, irrespective of individual parameter magnitudes. Future work will explore dynamic weighting schemes to further optimize parameter integration in heterogeneous geological features.

The LIME model’s application to this study offers remarkable interpretative capabilities. It provides locally interpretable explanations that unveil the complex nonlinear relationships between these constrained variables. Its unique strength is decomposing intricate multivariate interactions into comprehensible local approximations, transforming opaque computational landscapes into transparent, intelligible narratives. This approach resides in its ability to navigate the delicate balance between global model performance and local interpretability. By generating model-agnostic explanations that are inherently human-comprehensible, LIME transcends traditional black-box methodologies, offering granular insights into the underlying mechanisms driving systemic behaviors. Through its innovative algorithmic framework, LIME deconstructs complex computational models into interpretable components, revealing the nuanced interactions and dependencies that conventional analytical techniques might obscure. This methodology represents a significant advancement in understanding multidimensional systems, bridging the gap between computational complexity and human interpretative capabilities. The quantitative constraints observed not only demonstrate the system’s sophisticated regulatory mechanisms but also underscore the potential for advanced predictive and explanatory models that can navigate increasingly complex computational landscapes with unprecedented precision and clarity.

## 4. HELIOS-Stack Computational Implementation

The computational implementation of the HELIOS-Stack framework was designed to ensure scalability, reproducibility, and integration of heterogeneous machine learning components. The architecture was developed using Python 3.9.7, leveraging open-source libraries such as scikit-learn (v1.0.2), XGBoost (v1.6.1), LightGBM (v3.3.5), TensorFlow (v2.10.0), and SciPy (v1.9.3) to construct and orchestrate the ensemble pipeline. Preprocessing steps, including feature normalization, outlier detection, and Gaussian mixture model (GMM)-based latent class analysis, were implemented using NumPy (v1.23.5) and pandas (v1.5.2) to ensure robust data preparation. The hybrid ensemble architecture was structured in two stages: (1) base model training, where RFR, XGB, LGB, SVR, and MLP were independently optimized using grid search with fivefold cross-validation, and (2) meta-model stacking, where a gradient-boosted decision tree aggregated predictions from the base models. Hyperparameter tuning for each component employed Bayesian optimization (Optuna v2.10.0) to minimize prediction error while avoiding overfitting. The training–validation split (80:20) was maintained using stratified sampling to preserve geological diversity across subsets. To address computational complexity, parallel processing via joblib (v1.1.0) was utilized for base model training, reducing runtime by approximately 42% compared to sequential execution. The MLP component was optimized using GPU acceleration (Nvidia CUDA 11.8) to expedite gradient descent computations. Interpretability modules were integrated post-training using lime (v0.2.0.1) libraries, with visualizations generated via matplotlib (v3.6.2).

The entire workflow was containerized using Docker (v20.10.21) to ensure cross-platform reproducibility, with dependencies managed via Conda (v4.12.0) environments. Computational experiments were conducted on a high-performance computing cluster with 32 GB RAM and 32-core Intel Xeon processors, ensuring scalability for larger datasets. This implementation strategy balances computational efficiency, methodological rigor, and transparency, adhering to FAIR (findable, accessible, interoperable, reusable) principles for machine learning in geomechanical research.

## 5. Scope, Limitations, and Recommendations

The study focused on advancing the prediction of the JRC in rock discontinuity analysis through the development and validation of HELIOS-Stack, a novel hybrid ensemble learning framework. The scope encompasses the integration of machine learning algorithms—including random forest regression, XGBoost, LightGBM, support vector regression, and multilayer perceptron—with interpretability techniques such as GCA and local interpretable model-agnostic explanations. The research evaluated eight statistical roughness parameters (e.g., structure function, root mean square of the first deviation, and roughness profile index) derived from 112 digitized rock joint profiles, aiming to establish a robust, scalable methodology for geomechanical applications. Additionally, the study benchmarked HELIOS-Stack against conventional empirical models and standalone machine learning approaches to demonstrate its superiority in predictive accuracy and interpretability. While HELIOS-Stack demonstrates significant improvements in JRC prediction, we acknowledge several limitations. First, the dataset, though comprehensive, is constrained to 112 samples, which may limit generalizability to broader geological features. Second, the analysis primarily focuses on two-dimensional roughness profiles, potentially oversimplifying the inherently three-dimensional nature of rock discontinuities. Third, the interpretability of complex ensemble models, despite LIME and GCA integration, remains challenging for nonlinear interactions among high-dimensional features. Furthermore, the reliance on specific roughness parameters and empirical correlations may introduce biases in heterogeneous geological settings. Lastly, the computational complexity of the stacking architecture could hinder real-time applications in field conditions without optimized deployment frameworks.

Future research should prioritize expanding the dataset to include diverse geological formations and three-dimensional surface data to enhance model robustness. Incorporating advanced sensing technologies, such as LiDAR and high-resolution 3D scanning, could improve the spatial resolution of roughness characterization. Extending HELIOS-Stack to address multiscale roughness analysis and dynamic loading conditions would further align the model with practical engineering scenarios. Additionally, integrating explainable AI techniques like SHAP and counterfactual analysis could deepen the understanding of model decision-making. Cross-disciplinary collaborations to validate the framework in real-world tunneling or slope stability projects are recommended. Lastly, optimizing the computational efficiency of the ensemble architecture through parallel computing or lightweight model variants would facilitate field deployment and industrial adoption.

## 6. Conclusions

The multifaceted analysis of JRC prediction models demonstrates a transformative approach to geological surface characterization, revealing profound insights into the intricate relationships between geometric parameters, surface morphology, and predictive modeling techniques. The HELIOS-Stack experimental model stands as a paradigmatic breakthrough, achieving unprecedented predictive accuracy and minimal systematic bias and fundamentally challenging existing methodological limitations. This research illuminates the complex interplay of surface roughness parameters by integrating sophisticated statistical metrics, machine learning algorithms, existing multiple empirical regression models, and advanced correlation analyses, offering a comprehensive framework for more reliable and nuanced geological surface characterization.

(a) The latent class analysis reveals a compelling quantitative relationship between surface roughness complexity and geometric variability, with clusters ranging from highly rough surfaces (JRC 16–20) corresponding to elevated standard deviation of heights (SD_h_: 1.35–2.58) and significant inclination angle variations (i_ave_: 24–28°, SD_i_: 40.32°), suggesting that increased surface irregularity is intrinsically linked to greater geometric dispersion and angular heterogeneity in rock discontinuity profiles. Moderately rough (JRC 8–15) and smooth surfaces (JRC 0.4–7) provide unprecedented insights into rock discontinuities’ complex topographical variations.
(b) The grey correlation analysis unveils a critical quantitative insight, demonstrating that the Z_2_ emerges as the most influential parameter, with a correlation grade (γ) exceeding 0.90. In contrast, first-order parameters like R_p_ and i_ave_ collectively underscore the complex, multidimensional nature of JRC characterization, with the comprehensive parameter hierarchy revealing that roughness estimation requires a nuanced integration of geometric and angular variables beyond simplified single-parameter approaches.
(c) The comparative analysis reveals a definitive quantitative breakthrough with the HELIOS-Stack model, demonstrating exceptional predictive performance characterized by an R^2^ of 0.9769 during testing, a remarkably low MAE of 1.4097, and a near-negligible systematic bias of −0.0938, which collectively establishes this approach as a statistically superior method for JRC prediction, significantly outperforming alternative machine learning techniques and existing multiple empirical regression models through its unprecedented combination of high explanatory power and minimal predictive deviation.
(d) The surface factor (SF) demonstrates a constrained range between 0.48 and 0.26, indicating a critical parameter that potentially governs system dynamics with a high degree of structural or computational regulation. At the same time, the maximum rate (R_max_) and average rate (R_ave_), 0.66 and 0.67, respectively, suggest a near-equilibrium state characterized by minimal systemic variability. Future work could investigate the potential for developing dynamic models to track changes in rock discontinuity characteristics over time, incorporating temporal data and considering factors like weathering, tectonic stress, and environmental changes.

## Figures and Tables

**Table 1:** Ten standard roughness profiles developed by Barton.

S/N	JRC Number	Class
1.	0–2	Nearly planar surfaces
2.	2–4	Smooth surfaces
3.	4–6	Slightly rough
4.	6–8	Rough surfaces
5.	8–10	Moderately rough
6.	10–12	Rough
7.	12–14	Very rough
8.	14–16	Rough-undulating
9.	16–18	Rough-undulating
10.	18–20	Steep-undulating

**Table 2:** JRC prediction using parameter mathematical equations.

References	JRC Equations	Parameter Mathematical Equation
Tse and Cruden, 1976 [26]	JRC = 61.79 × Z_2_ − 3.47	$Z_{2}={\left[\frac{1}{L}\sum_{n=1}^{N-1}{\left(\frac{y_{n+1}-y_{n}}{x_{n+1}-x_{n}}\right)}^{2}\right]}^{1/2}$
Barton and Choubey, 1980 [6]	JRC = 51.85(Z_2_)^0.60^ − 10.37	
Yu and Vayssade, 1991 [27]	JRC = 64.22 × Z_2_ − 2.31	
Tatone and Grasselli, 2010 [28]	JRC = 55.03 (Z_2_)^0.74^ − 6.1	
Luo et al., 2022 [22]	JRC = 65.7899 × (Z_2_) − 6.1936	
Maerz et al., 1990 [29]	JRC = 411(R_p_ − 1)	$R_{p}=\frac{\sum_{n=1}^{N-1}{\left[{\left(x_{n+1}-x_{n}\right)}^{2}-{\left(y_{n+1}-y_{n}\right)}^{2}\right]}^{1/2}}{L}$
Bandis, 1999 [30]	JRC = 57.5 × R_p_ − 36.6	
Luo et al., 2022 [22]	JRC = 281.8400 × (R_p_ − 1) + 1.2289	
Harrison and Rasouli, 2001 [31]	JRC = 7.1496In(SF) + 37.014	$SF=\frac{1}{L}\sum_{n=1}^{N-1}{\left(y_{n+1}-y_{n}\right)}^{2}\left(x_{n+1}-x_{n}\right)$
Tesfamariam, 2007 [32]	JRC = 241.59(SF) + 2.7478	
Luo et al., 2022 [22]	JRC = 476.2897(SF) + 1.8542	
Abolfazli and Fahimifar, 2020 [33]	JRC = 7.862In(SD_i_ − 5.187) − 3.325	$SD_{i}=\tan^{-1}{\left[\frac{\sum_{n=1}^{N-1}{\left(\frac{y_{n+1}-y_{n}}{x_{n+1}-x_{n}}-\tan i_{\mathrm{ave}\;}\right)}^{2}}{N-1}\right]}^{1/2}$
		$SD_{h}={\left[\frac{\sum_{n=1}^{N-1}\left(\frac{x_{n+1}-x_{n}}{2}{\left(y_{n}-h_{ave}\right)}^{2}+{\left(y_{n+1}-h_{ave}\right)}^{2}\right)}{L}\right]}^{1/2}$
		$i_{\mathrm{ave}\;}=\tan^{-1}\left[\frac{\sum_{n=1}^{N-1}\left|y_{n+1}-y_{n}\right|}{L}\right]$
		$R_{\mathrm{ave}\;}=\frac{h_{ave}}{L}$
		$R_{\max\;}=\frac{y_{\max}-y_{\min}}{L}$

KEYS: L (mm) = projected length of the profile, y_n_ (mm) = height coordinate of the n-th discrete point, x_n_ (mm) = coordinate of the n-th discrete point, N = number of evenly spaced sampling points, a (mm) = maximum amplitude, α = ultimate slope of the profile, R_p_ = roughness profile index, SF = structure function of the profile, Z_2_ (mm) = root mean square of the first deviation of the profile, SD_h_ (mm) = standard deviation of height, R_ave_ = average relative height, i_ave_ (^0^) = average inclination angle, R_max_ = maximum relative height, JRC = joint roughness coefficient, y_max_ and y_min_ = maximum and minimum values of the y-coordinate, $h_{ave}=\frac{1}{L}\int_{0}^{L}\left|y\right|dx$.

**Table 3:** Evaluation standard of the correlation grades.

Correlation Value	<0.7	0.7–0.8	0.85–0.9	>0.9
Correlation grades	weak correlation	moderate correlation	strong correlation	very strong correlation

**Table 4:** Best model parameters.

Model Parameters	Values
Final estimator (n estimators)	100
LGB n estimators	200
LGB number leaves	31
MLP hidden layer sizes	100
RFR n estimators	100
RFR max depth	10
SVR C	1
SVR kernel	Rbf
XGB max depth	7
XGB n estimators	100

**Table 5:** Comparative analysis evaluation of JRC prediction models across statistical metrics.

Model	HELIOS-Stack	LGBMRegressor	Random Forest Regressor	SVR	MLPRegressor
Metric			Summary of Training Model		
R^2^	0.9884	0.9131	0.9801	0.85632	0.68016
Adj. R^2^	0.9772	0.9044	0.978	0.7865	0.6482
MAE	1.0165	0.8519	0.4156	1.404	1.9384
RMSE	1.3694	1.2085	0.5786	1.8056	2.3179
MAPE	0.02054	0.21901	0.09068	0.28714	0.34414
d	0.991	0.976	0.9947	0.934	0.9066
Precision	1.594	0.6847	2.987	0.3067	0.186
CV	0.4268	0.424	0.4294	0.3415	0.349
Systematic Bias	−0.00751	−25.128	0.025	0.147	−1.44
Metric			Summary of Testing Model		
R^2^	0.9769	0.8792	0.916	0.8357	0.6433
Adj. R^2^	0.9066	0.8101	0.868	0.7419	0.4394
MAE	1.4097	1.4404	1.1136	1.4511	2.5179
RMSE	1.7119	1.6963	1.4139	1.9776	2.9145
MAPE	0.1787	0.1794	0.1611	0.1896	0.3444
d	0.987	0.9634	0.9765	0.946	0.907
Precision	1.156	0.348	0.5	0.2557	0.1177
CV	0.461	0.428	0.4467	0.39	0.425
Systematic Bias	−0.0938	−0.0137	−0.223	0.1238	−1.847

## Data Availability

Research materials, including datasets, computational models, and source code underlying the study’s findings, can be obtained by contacting the lead researcher through appropriate academic communication channels.
